# Phenotypic, physicochemical, sensory, and functional attributes of cocoa (*Theobroma cacao* L.) from Brazilian Amazonian floodplain and upland ecosystems

**DOI:** 10.1002/jsfa.70793

**Published:** 2026-06-10

**Authors:** Danielle Amaral e Silva, Consuelo Lúcia Sousa de Lima, Giulia Victória Silva Lima, Angela Maria Miranda Silva, Neirivaldo Cavalcante da Silva, Hervé Louis Ghislain Rogez, Jesus Nazareno Silva de Souza

**Affiliations:** ^1^ Graduate Program in Food Science and Technology Institute of Technology, Federal University of Pará Belém Brazil; ^2^ Center for Valorization of Amazonian Bioactive Compounds (CVACBA) Federal University of Pará (UFPA) Belém Brazil; ^3^ Graduate Program in Science and Environment Institute of Natural and Exacts Science, Federal University of Pará Belém Brazil

**Keywords:** Amazonian cocoa, floodplain cocoa, bioactive compounds, sensory profile

## Abstract

**BACKGROUND:**

Cocoa (*Theobroma cacao* L.) from the Brazilian Amazon plays an important role in national production. While most cocoa is cultivated in upland systems, floodplain ecosystems represent a traditional and distinct production environment that may influence bean composition and quality. This study characterizes floodplain cocoa (FC) and compares it with upland cocoa (UC) from the eastern Amazon.

**RESULTS:**

FC fruits had an oval shape and thick husks, with smaller beans than UC, both well fermented. FC beans had higher fiber content (17.70 ± 0.7 g/100 g dry matter (DM)) and total polyphenols (27.04 ± 0.69 mg CE/g DM), but lower protein content (14.99 g/100 g DM) and antioxidant capacity (627.45 ± 30.99 μmol TE/g DM) compared to UC beans (fiber: 14.57 ± 0.40 g/100 g DM; protein: 16.44 g/100 g DM; polyphenols: 14.19 ± 4.19 mg CE/g DM; antioxidant capacity: 1112.58 ± 56.47 μmol TE/g DM). FC beans showed higher levels of caffeine and theophylline, and a theobromine/caffeine ratio typical of Criollo cocoa. Chocolates from both exhibited favorable attributes, including fine‐flavor notes, and were similarly accepted by consumers.

**CONCLUSION:**

FC and UC beans exhibited distinct physicochemical profiles, both showing adequate fermentation and similar consumer acceptance, highlighting the potential of Amazonian cocoa for product differentiation and valorization of regionally distinct systems. © 2026 The Author(s). *Journal of the Science of Food and Agriculture* published by John Wiley & Sons Ltd on behalf of Society of Chemical Industry.

## INTRODUCTION

Cocoa (*Theobroma cacao* L.) is a highly valued crop due to its beans, which are used in the production of chocolate, beverages, ice creams, and a variety of desserts.^1^ While cocoa products are primarily processed and consumed in industrialized countries, the crop is cultivated mainly in tropical developing regions such as West Africa, Latin America, and Asia.^2^ In Brazil, cocoa has gained significant prominence in agribusiness, positioning the country as the seventh‐largest cocoa producer globally. In 2023, national production reached 296 145 tons of cocoa beans, with the state of Pará, located in the Amazon region, leading production. Pará accounts for 96% of cocoa output in Brazil's northern region and 47% of the national total.^3^

Traditionally, cocoa has been classified into three primary cultivars: Criollo, Trinitario, and Forastero. Cocoa populations from the Amazon basin are generally grouped under Forastero, recognized for their disease resistance and high productivity.^4^ In 2008, however, Motamayor *et al*.^5^ proposed a new classification of cocoa germplasm into ten major groups – Marañon, Curaray, Criollo, Iquitos, Nanay, Contamana, Amelonado, Purús, Nacional, and Guiana – to better reflect the genetic diversity within the species.

Although most cocoa produced in the Amazon is cultivated on upland soils, cocoa is tolerant to flooding and has been grown along Amazonian floodplains for centuries. It is estimated that approximately 9500 ha of floodplain areas in the states of Pará and Amazonas are covered by ‘wild’ cocoa trees.^6^ Cocoa producers in Amazonian floodplains are primarily composed of riverine communities, and quilombolas, representing traditional forest peoples. These groups sustainably exploit cocoa through extractive practices, often in association with other useful species, contributing significantly to the local economy through job creation and income generation.^7^, ^8^

The chemical composition of floodplain soils is highly variable and influenced by multiple factors, including local geology, the type and density of vegetation, the hydrological characteristics of the river, and the frequency and duration of flooding events. In general, these soils exhibit high water retention capacity and are often richer in nutrients compared to upland soils of the Amazon region.^8^, ^9^ As a result of these unique edaphic conditions, cocoa beans cultivated in floodplain ecosystems may develop distinctive attributes, including aromatic and flavor profiles that enhance bean quality.^9^, ^10^

The interaction of intrinsic and extrinsic factors plays a crucial role in balancing the compounds that define the sensory profile of cocoa. The quality of cocoa beans is determined by a combination of factors, including their nutritional composition and postharvest management practices. While fats, proteins, and carbohydrates make up the primary components of cocoa beans, they also contain secondary metabolites such as phenolic compounds and methylxanthines, which contribute to the bitterness and astringency of raw beans.^11^ Among the phenolic compounds found in cocoa, flavan‐3‐ols like epicatechin and catechin are predominant, while theobromine, caffeine and theophylline are the primary methylxanthines.^12^ These bioactive compounds are of great interest in the nutraceutical industry due to their health‐promoting properties.^13^

Given the limited scientific literature regarding the characterization of floodplain cocoa (FC) from the eastern Amazon, this study aims to characterize cocoa produced in floodplain ecosystems based on its physical, physicochemical, functional, and sensory attributes, and to compare it with beans cultivated in upland ecosystems. Identifying and understanding these characteristics may contribute to product differentiation and enhance the market value of Amazonian cocoa, thereby strengthening its competitiveness in both domestic and international markets.

## MATERIALS AND METHODS

### Cocoa fruit and beans acquisition

Cocoa fruits from floodplain ecosystems were collected during the 2021 and 2022 harvests for physical characterization from three locations: Ilha do Combu, Belém (1°30′11.5″ S, 48°26′48.8″ W; 1°29′41.5″ S, 48°29′17.0″ W; 1°29′35.5″ S, 48°28′37.1″ W), Acará (2°06′04.4″ S, 48°29′16.1″ W; 2°06′07.0″ S, 48°31′28.9″ W; 2°06′06.8″ S, 48°31′24.2″ W), and Moju (1°46′43.9″ S, 48°35′27.7″ W; 1°46′20.4″ S, 48°35′32.8″ W; 1°46′45.8″ S, 48°35′29.8″ W). In addition, commercial cocoa beans were acquired directly from local producers in these same municipalities (three producers per municipality, totaling nine samples). These samples were pooled and are referred to as FC for this study.

For comparison, an equal number of commercial cocoa beans were also acquired from producers in upland ecosystems in the municipalities of Moju (2°06′02″ W, 48°40′05″ S), Uruará (3°35′10″ W, 53°24′45″ S), Altamira (3°08′08″ W, 52°13′24″ S), Medicilândia (53°9′6.897″ W, 3°8′53.533″ S), and Tomé‐Açu (2°24′37.4″ S, 48°10′15.3″ W). These samples were combined and designated as UC (upland cocoa).

Fermentation and drying were carried out by the producers prior to sample collection, following their traditional post‐harvest practices. Despite differences between cultivation ecosystems, post‐harvest practices were generally similar among producers. According to information provided by the producers at the time of sampling, fermentation was carried out spontaneously on farms using wooden boxes, and drying was performed by sun exposure, with beans spread on tarpaulins or drying platforms (barcaças). The beans from both ecosystems had an average fermentation time of 6.67 ± 0.58 days and an average drying time of 6.33 ± 0.67 days. All samples were transported at room temperature to the laboratory at the Federal University of Pará (UFPA), Belém, Brazil, where they were stored at −4 °C for subsequent analyses.

### Physical characterization of floodplain cocoa

**Figure 1:**
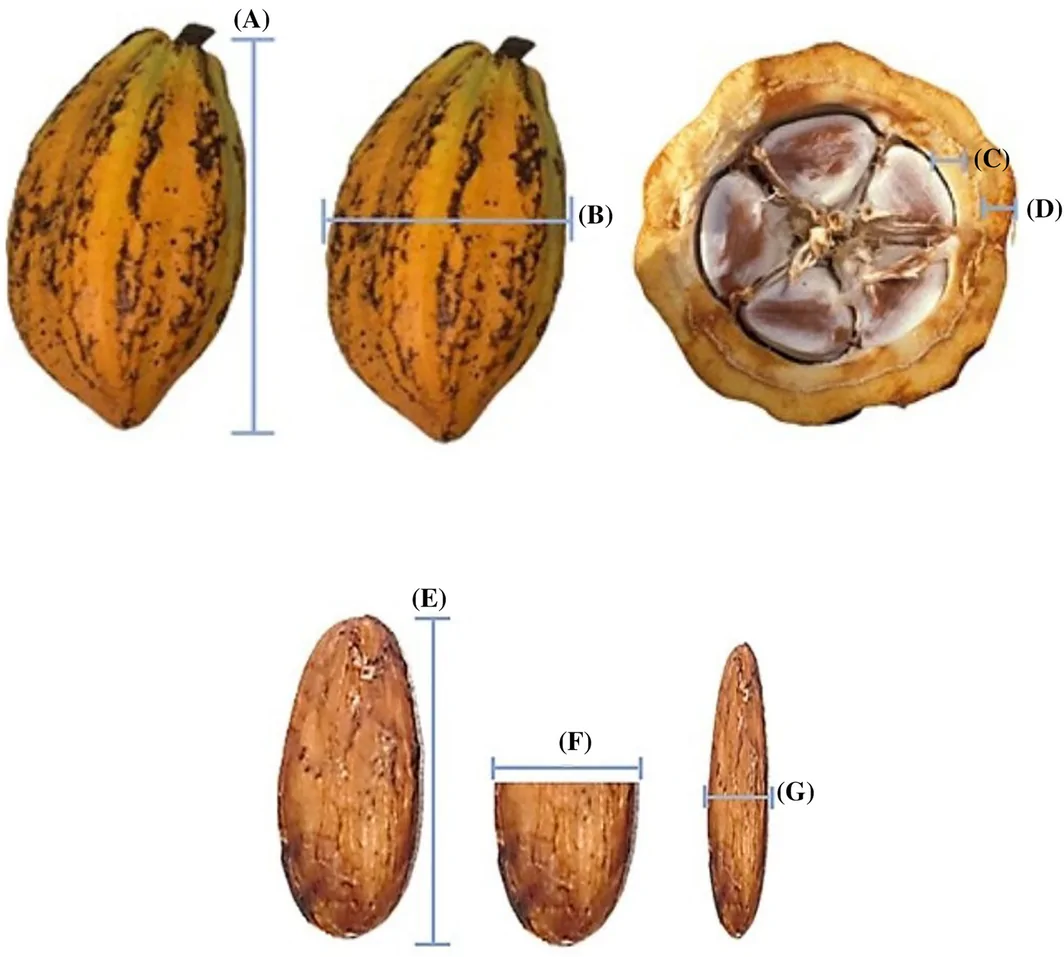
Morphometric parameters assessed in cocoa fruit and seeds, including longitudinal (A) and transverse diameters of the fruit (B); inner (C) and outer shell thickness (D); longitudinal (E) and transverse diameters of the seed (F); seed thickness (G).

The FC fruits were washed with running water and sanitization with chlorinated water (200 ppm). Subsequently, external and internal physical measurements (husk and beans) were performed using a caliper and an analytical balance. The morphometric parameters evaluated included: total fruit mass (TFM), longitudinal diameter (LD) and transverse diameter (TD) of the fruit; LD/TD ratio; husk thickness, measured at two points: the central region of the inner husk thickness (IHT) and the central region of the outer husk thickness (OHT); OHT/IHT ratio; total husk mass (THM); number of seeds (NS); seed mass (SM); seed thickness (ST); seed transverse diameter (STD) and seed longitudinal diameter (SLD); SLD/STD ratio (Fig. 1).

### Evaluation and characterization of fermented and dried cocoa beans

FC and UC cocoa beans samples were analyzed using the bean count method, in which beans were weighed until reaching 100 g, and the average weight per bean was calculated. A cut test was performed on 300 beans, which were longitudinally cut to assess the degree of fermentation based on color (brown, partially brown, violet) and cotyledon compartmentalization.^14^, ^15^ After manual peeling, the nibs were ground (< 1 mm) and stored at −18 °C.

The pH was measured in an aqueous suspension (5 g of cocoa nibs in 45 mL of water) using a digital pH meter. Titratable acidity was determined by potentiometric titration with sodium hydroxide (0.1 mol/L). Water activity was assessed by measuring water fugacity (AQUALAB model; Decagon Devices, Pullman, WA, USA). Proximate composition was determined according to AOAC methodologies, with results expressed in grams per kilogram of dry matter (g/kg DM).^16^ Moisture content was measured by drying the samples at 105 °C for 24 h. Total lipids were extracted with petroleum ether using a Soxhlet apparatus, protein content was determined using the Kjeldahl method (conversion factor of 6.38), ash content was obtained by incineration at 550 °C, and crude fiber was determined by acid and alkaline digestion. Total carbohydrates were calculated by difference. All analyses were performed in triplicate.

### Analysis of bioactive compounds and antioxidant capacity

Bioactive compounds were extracted from 10 g of cocoa nibs using three successive extractions with 50 mL of an acetone–water–acetic acid mixture (70:29.5:0.5, *v/v/v*) under constant agitation at 25 °C for 1 h.^17^ The extracts were then homogenized, filtered through Whatman™ paper, and used for further analyses.

Total polyphenol content was determined using the Folin–Ciocalteu method, with absorbance measured at 735 nm using a spectrophotometer (Biotrak, Molecular Devices, Sunnyvale, CA, USA). Results were expressed as milligrams of catechin equivalent per gram of dry matter (mg CE/g DM).^18^ Antioxidant capacity was determined using the oxygen radical absorbance capacity (ORAC) assay in a 96‐well microplate based on the protocol proposed by Huang *et al*.^19^ Fluorescence was measured using a microplate fluorescence reader (Bio‐Tek Instruments, Winooski, VT, USA). Results were expressed as micromoles of Trolox equivalent per gram of dry matter (μmol TE/g DM).

The quantification of major phenolic compounds ((+)‐catechin and (−)‐epicatechin) and methylxanthines (caffeine, theobromine, and theophylline) was performed based on the work of Lima *et al*.^20^ Analyses were carried out on an Ultimate 3000 ultrahigh‐performance liquid chromatography (UHPLC) system (Thermo Scientific, Waltham, MA, USA) equipped with a quaternary pump (LPG‐3400 RS), an automatic injector (WPS‐3000SL Analytical), a flow cell (Standard Analytical), a diode‐array detector (DAD, DAD‐3000), and Chromeleon 7.1 SR2 software. A Kinetex C18 2.6 μm 100 mm × 4.6 mm column (Phenomenex, Torrance, CA, USA) was used at 35 °C, using water (solvent A) and acetonitrile (solvent B), both acidified with 2.5% acetic acid, as mobile phases in a programmed gradient. The elution gradient started at 5% B and increased to 25% B over 9 min, then to 95% B until 12.5 min, remaining constant until 15 min. Then, it returned to 5% B until 18 min for the initial system conditions. Chromatograms were recorded between 200 and 800 nm, and the compounds were quantified at 280 nm, except for procyanidin B2, which was quantified at 233 nm. A flow rate of 1 mL/min was applied and 20 μL of the extracts were injected into the UHPLC system. All analyses were performed in triplicate, and the results were expressed in milligrams per gram of dry matter (mg/g DM).

### Chocolate production

Chocolates were produced from FC and UC samples using a formulation of 70% cocoa nibs and 30% sugar, without the addition of emulsifiers or flavoring agents.^11^ Approximately 1 kg of cocoa beans were roasted at 120 °C for 60 min, followed by manual separation of nibs and shells. The nibs were ground in a blender (900 W, 60 Hz; Philco, Philadelphia, PA, USA) and subsequently refined for 24 h in a stone mill (Premier, Chocolab, São Paulo, SP, Brazil) with the addition of sugar. The chocolate mass was then tempered to stabilize the crystalline structure of cocoa butter, following a three‐step process: heating to 45–50 °C, cooling to 27–28 °C, and reheating to 31–32 °C. Finally, the chocolates were molded and stored at −10 °C until sensory analysis.

### Quantitative descriptive analysis

Sensory analyses were performed using the quantitative descriptive analysis (QDA) method,^21^ after approval by the Research Ethics Committee of the Federal University of Pará (approval number 4.031.506).

A sensory panel consisting of 13 assessors was recruited and trained to identify and quantify cocoa sensory attributes according to ISO 8586:2023.^22^ Panel training was conducted continuously over a 6‐month period. Descriptors were defined using reference materials, including standard solutions (e.g., caffeine for bitterness and citric acid for acidity) and representative food/aroma references. The definition, application, and validation of these descriptors were performed in accordance with the guidelines established by ISO 13299:2016.^23^

The final evaluation was performed using a ten‐point structured scale to measure attribute intensity, where 0 = not perceptible; 0.5–2.0 = barely perceptible; 2.5–5.0 = moderate; 5.5–6.5 = moderately high; 7.0–8.0 = strong/high; and 8.5–10.0 = very strong/very high. Chocolate samples were presented monadically in sequential order using randomized three‐digit codes. Palate cleansing between samples was performed using water and unsalted crackers. The tests were conducted in triplicate in individual sensory booths under white lighting conditions at the Sensory Analysis Laboratory (UFPA).

### Acceptance test

An acceptance test was performed with 50 untrained consumers of dark chocolate, with no restrictions regarding age, gender, or socioeconomic status. The participants were regular chocolate consumers (≥ 1 time/week), and individuals with dietary restrictions related to cocoa were excluded from the study. The panelists evaluated chocolates produced from FC and UC samples using a structured nine‐point hedonic scale. The attributes assessed included aroma, flavor, bitterness, acidity, texture, astringency, and overall impression. The scale ranged from 1 (‘disliked it very much’) to 9 (‘liked it very much’), with the following anchors: 9 (‘liked it very much’), 8 (‘liked it a lot’), 7 (‘liked it moderately’), 6 (‘liked it slightly’), 5 (‘neither liked nor disliked’), 4 (‘disliked it slightly’), 3 (‘disliked it moderately’), 2 (‘disliked it much’), and 1 (‘disliked it very much’).

### Data analysis

Analysis of variance (ANOVA) and the Tukey test were conducted to determine differences between the means at a significance level of 95%. The results were statistically processed using STATISTIC 7.0 software.

## RESULTS AND DISCUSSION

### Physical attributes of cocoa fruits from floodplain ecosystems

**Figure 2:**
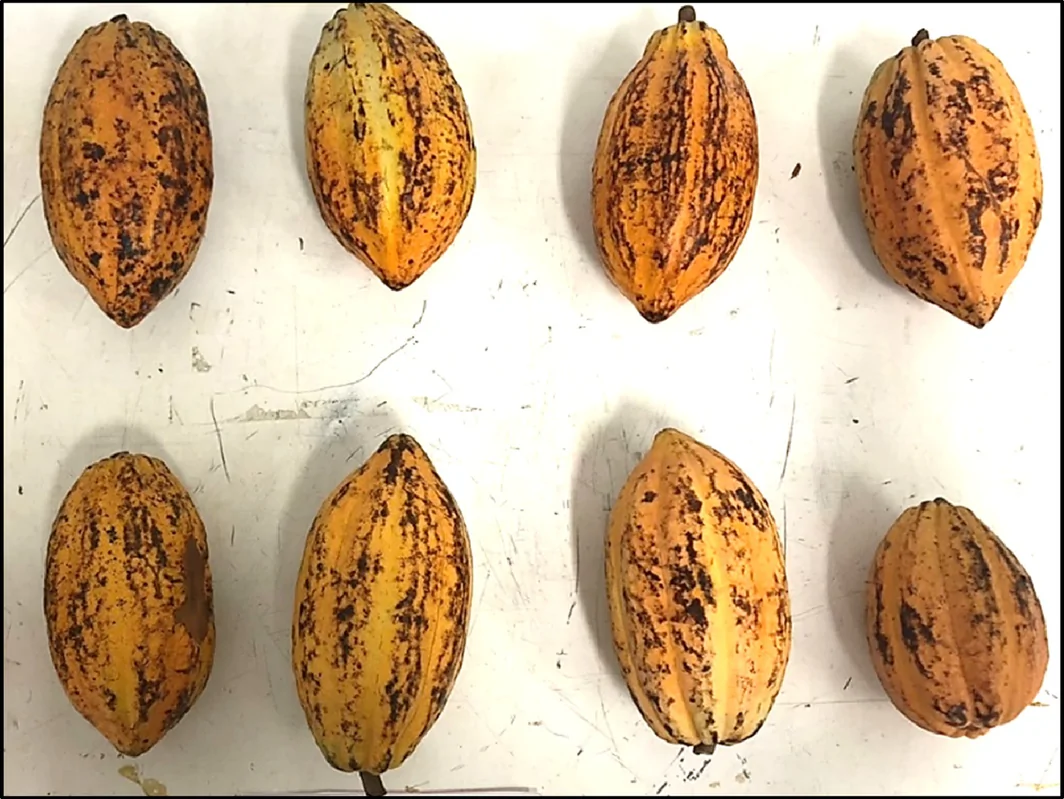
Appearance of floodplain cocoa fruits from the Brazilian Amazon.

The FC fruits (*n* = 90) collected had an average TFM of 377.04 ± 39.80 g, a LD of 14.56 ± 0.95 cm, a TD of 7.66 ± 0.27 cm, and an LD/TD ratio of 1.94 ± 0.09, indicating an oval‐shaped profile. This shape is typical of Forastero cocoa, which is known for its short and oval fruits (Fig. 2). In contrast, Criollo and Trinitario varieties tend to produce more elongated fruits with LD exceeding 20 cm.^24^ The shape of cocoa fruits is influenced by a combination of genetic heritability and external factors, including soil composition, climatic conditions, and agricultural practices.^25^ TFM is primarily determined by size but is also influenced by shape, as more oval fruits tend to exhibit denser tissue distribution. UC fruits from the same region have shown a wide range of TFM, varying from 236.4 to 702.6 g,^12^ reflecting the variability in these ecosystems.

The husk parameters showed an average THM of 281.8 ± 35.97 g, OHT of 1.83 ± 0.36 cm, IHT of 0.56 ± 0.06 cm, and an OHT/IHT ratio of 3.26 ± 0.23. The fruit husk accounted for approximately 75% of the fruit's total mass, similar to what has been reported for UC fruits.^26^ The OHT and OHT/IHT ratio indicate a robust husk in FC fruits. Thicker outer layers provide additional barriers against external agents, such as *Phytophthora palmivora*, one of the leading causes of cocoa production losses.^27^ This characteristic enhances resilience to environmental and biological challenges, making it a promising attribute for commercial cocoa production.

FC fruits exhibited an average NS of 33.96 ± 2.58 per fruit with an average SM of 82.73 ± 4.41 g/fruit, a ST of 2.96 ± 0.26 cm, a STD of 1.12 ± 0.03 cm, a SLD of 2.12 ± 0.13 cm, and an SLD/STD ratio of 1.89 ± 0.08. These dimensional characteristics suggest that the seeds had a flat profile and an oval shape. The NS is influenced by several factors, including the number of ovules per ovary, the fertility of the ovules – which depends on the self‐compatibility or self‐incompatibility of the cocoa tree genotype – and natural pollination conditions. UC fruits with similar TFM also exhibit comparable NS values.^25^ SM for the same clone can vary across different regions due to climatic and cultivation differences, including soil characteristics.^25^

The characterization of cocoa from floodplain ecosystems is essential to expand the database on these fruits. While morphological characterization is a valuable tool, it should not be used as a definitive method for identification but rather as a complementary component of a broader approach to germplasm classification.

### Physical and physicochemical characteristics of fermented and dried cocoa beans

**Figure 3:**
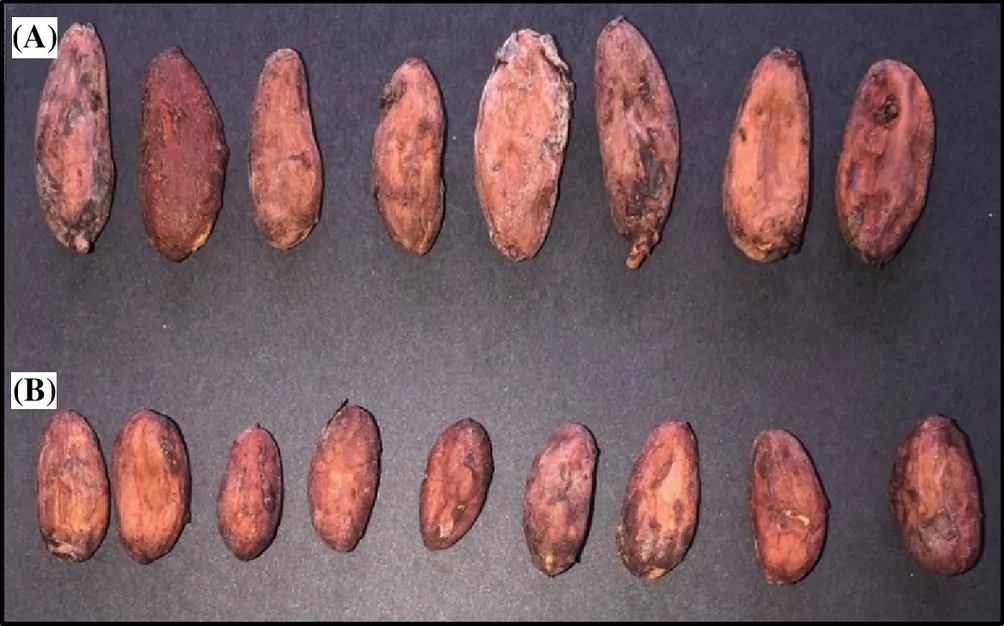
Appearance of cacao beans from the Brazilian Amazon ecosystems: (A) upland; (B) floodplain.

Table 1 presents the physical characteristics of FC and UC beans. The bean count parameter is used by the Federation of Cocoa Commerce (FCC) to classify cocoa beans into four categories: standard beans (< 1000 beans/kg), medium beans (1001–1100 beans/kg), small beans (1101–1200 beans/kg), and very small beans (>1200 beans/kg).^28^ According to this classification, UC beans fall into the standard bean category, while FC beans are classified as medium beans (Fig. 3). The FC beans have a higher average mass compared to UC beans (*P* < 0.01), confirming that FC cocoa beans are smaller in size.

**Table 1:** Physical characteristics of floodplain cocoa (FC) beans and upland cocoa (UC) beans obtained through weighing and cut test

	FC	UC	*P*‐Value
Bean count (beans/kg)	1039.6 ± 23.0^a^	952.2 ± 29.0^b^	<0.001
Average mass (g/bean)	0.98 ± 0.03^b^	1.10 ± 0.04^a^	<0.01
Brown (%)	65.00 ± 3.76^a^	65.98 ± 1.19^a^	0.718
Partially brown (%)	30.13 ± 2.39^a^	29.05 ± 1.66^a^	0.584
Violet (%)	4.87 ± 0.45^a^	4.97 ± 0.13^a^	0.103
Compartmentalized (%)	98.10 ± 4.43^a^	90.40 ± 5.11^b^	<0.001
Uncompartmentalized (%)	1.90 ± 5.12^a^	9.59 ± 4.68^b^	<0.001

*Note*: Results expressed as mean ± standard deviation. Different letters in the same line indicate statistical differences (one‐way ANOVA and Tukey's test, *P* < 0.05).

The cut test assesses the degree of fermentation in cocoa beans by analyzing the color and the compartmentalization of the cotyledons. During fermentation, biochemical reactions result in browning of the beans. For the Forastero genetic group, well‐fermented beans are characterized by more than 65% of the cotyledons displaying a brown color, no more than 15% showing violet coloration, and clear compartmentalization. Violet beans are often associated with incomplete fermentation.^20^

Beans from both FC and UC samples exhibited similar results in the cut test, with approximately 5% classified as violet, 65% as brown. Although a statistical difference was observed for compartmentalization, both samples presented values above 90%, indicating a high degree of proper compartmentalization. These findings align with the expected outcomes for beans with an average fermentation duration of 6 days. Lima *et al*.^20^ reported that a minimum fermentation period of 4 days is necessary for cocoa beans from the Brazilian Amazon to achieve favorable cut test scores. Nonetheless, fermentation practices among producers in Pará exhibit variability, with durations ranging from 4.1 to 6.8 days.^9^ Proper fermentation is a critical determinant of high‐quality chocolate, contributing to the development of its desirable flavor profile.^29^

The physicochemical characteristics of FC and UC beans were also analyzed, including pH, water activity, titratable acidity, proximate composition (moisture, protein, lipids, ash, fiber, and carbohydrates), and energy value. The results are summarized in Table 2.

**Table 2:** Physical and chemical characteristics of floodplain cocoa (FC) beans and upland cocoa (UC) beans from ecosystems of the Brazilian Amazon

Characteristic	FC	UC	*P*‐Value
pH	5.43 ± 0.21^a^	5.07 ± 0.18^b^	<0.001
Titratable acidity (g acetic acid/kg)	3.5 ± 0.30^a^	3.7 ± 0.40^a^	0.302
Water activity	0.62 ± 0.05^a^	0.56 ± 0.03^a^	0.139
Moisture (g/kg)	71.0 ± 3.7^a^	59.8 ± 0.9^b^	<0.001
Total proteins (g/kg)	149.9 ± 1.9^b^	164.4 ± 2.7^a^	0.004
Ash (g/kg)	26.1 ± 0.8^a^	22.3 ± 3.0^a^	0.841
Crude fiber (g/kg)	177.0 ± 7.0^a^	145.7 ± 4.0^b^	0.006
Total lipids (g/kg)	351.0 ± 36.0^a^	357.1 ± 19.0^a^	0.227
Carbohydrates (g/kg)	308.9 ± 25.9^a^	329.8 ± 14.6^a^	0.371
Energy value (kcal/g)	4522.0 ± 187.9^a^	4822.2 ± 71.3^a^	0.204

*Note*: Results expressed as mean ± standard deviation. Different letters in the same line indicate statistical differences (one‐way ANOVA and Tukey's test, *P* < 0.05).

Both UC and FC cocoa beans showed similar titratable acidity and water activity. Although a statistical difference was observed between the samples, the pH values of both fell within the expected range for well‐fermented beans (between pH 5 and pH 5.5), which favors protease activity and promotes protein degradation, leading to the formation of free amino acids that act as precursors of aromatic compounds.^30^

Acidity is also a key parameter in defining the flavor, aroma, and texture of cocoa products. In the present study, the acidity of fermented and dried beans was 3.5 ± 0.3 g acetic acid/kg for FC and 3.7 ± 0.4 g acetic acid/kg for UC, with no statistically significant differences. Penido *et al*.^31^ reported an acidity of 28.8 g acetic acid/kg in Brazilian cocoa nibs immediately after fermentation. Excessive residual acidity can result in a pronounced acidic taste, compromising the characteristic chocolate aroma of the products.

The water activity and moisture parameters highlight the efficiency of the drying process for the cocoa beans. The water activity values were 0.62 ± 0.05 for UC and 0.56 ± 0.03 for FC. Water activity is critical for inhibiting microbial growth, particularly of mycotoxin‐producing fungi such as *Aspergillus ochraceus* and *Aspergillus parasiticus*, which require a minimum water activity of 0.78 to grow and 0.85 to produce mycotoxins.^27^, ^32^ Despite statistical differences in moisture content (71.0 ± 3.7 g/kg in FC and 59.8 ± 0.9 g/kg in UC), both values fall within the range recommended, ensuring safe transport and commercialization of the beans.^14^

Regarding the proximate composition, significant differences were observed only in the protein and fiber contents of the cocoa beans. FC beans had a higher fiber content (177.0 ± 7.0 g/kg *versus* 145.7 ± 4.0 g/kg in UC), while UC beans exhibited higher protein content (164.4 ± 2.7 g/kg *versus* 149.9 ± 1.9 g/kg in FC). The fiber content of cocoa beans is known to vary depending on the variety, reflecting the influence of genetic factors.^33^

Despite the environmental differences between ecosystems, the protein content of Amazonian cocoa beans is comparable to that of beans from Côte d'Ivoire (157 g/kg), and higher than those reported for the Dominican Republic (118 g/kg) and Cameroon (ranging from 117 to 133.5 g/kg).^33^, ^34^ Typically, proteins constitute 10–15% of the dry weight of cocoa beans. In addition to the genotype and origin of the beans, protein composition is heavily influenced by factors such as the plant's defense mechanisms, exposure to stress, photosynthesis efficiency, and hormonal metabolism during the growth of the cocoa tree.^35^

The other physicochemical parameters are consistent with those previously reported for cocoa beans from the Brazilian Amazon.^9^, ^11^, ^36^

### Bioactive compounds and antioxidant capacity of the cocoa beans

UC and FC beans were analyzed for total polyphenols and antioxidant capacity. In addition, the main phenolic compounds ((−)‐epicatechin, (+)‐catechin, and procyanidin B2,) and methylxanthines (theobromine, caffeine, and theophylline) in cocoa were quantified using HPLC‐DAD. The results are presented in Table 3.

**Table 3:** Bioactive compounds and antioxidant capacity of floodplain cocoa (FC) beans and upland cocoa (UC) beans from ecosystems of the Brazilian Amazon

	FC	UC	*P*‐Value
Total polyphenols (mg CE/g DM)^†^	27.04 ± 0.69^a^	14.19 ± 4.19^b^	<0.001
Antioxidant capacity (μmol TE/g DM)^‡^	627.45 ± 30.99^b^	1112.58 ± 56.47^a^	0.011
Epicatechin (mg/g)	1.72 ± 0.00^b^	3.32 ± 1.10^a^	<0.001
Catechin (mg/g)	0.32 ± 0.02^a^	0.33 ± 0.17^a^	0.065
Procyanidin B2 (mg/g)	3.31 ± 0.13^a^	0.95 ± 0.22^b^	<0.001
Theobromine (mg/g)	4.72 ± 0.23^a^	5.40 ± 2.61^a^	0.003
Caffeine (mg/g)	2.65 ± 0.14^a^	1.16 ± 0.04^b^	<0.001
Theophylline (mg/g)	0.41 ± 0.06^a^	0.20 ± 0.04^b^	<0.001

*Note*: Results expressed as mean ± standard deviation. Different letters in the same line indicate statistical differences (one‐way ANOVA and Tukey's test, *P* < 0.05).

^†^ Milligrams of catechin equivalent per gram of dry matter.

^‡^ Micromoles of Trolox equivalent per gram of dry matter.

The total polyphenol analysis revealed a significantly higher content in FC beans (27.04 mg CE/g DM) compared to UC beans (14.19 mg CE/g DM). This value encompasses not only the flavan‐3‐ols (catechins and procyanidins) mentioned earlier but also other classes of polyphenols, such as flavonols, flavanones, and flavones. Additionally, plants produce oligomeric and polymeric forms of polyphenols, such as pro‐anthocyanidins derived from flavan‐3‐ols.^37^ This may be attributed to environmental stressors typical of floodplain ecosystems, such as periodic flooding and soil variability, which are known to stimulate the biosynthesis of secondary metabolites like polyphenols.^38^

In contrast, UC beans demonstrated higher antioxidant capacity (1112.58 μmol TE/g DM *versus* 627.45 μmol TE/g DM in FC). This suggests that, despite the higher total polyphenol content in UC beans, the bioactive compounds in UC beans may be more efficient in terms of antioxidant activity. Similar variations in epicatechin content (ranging from 1.17 to 3.17 mg/g) have already been reported for Amazonian cocoa beans from different municipalities, highlighting the influence of regional and environmental factors on the bioactive compound profile.^20^

In contrast, FC beans had higher levels of procyanidin B2, a dimeric flavanol whose antioxidant activity is dependent on its degree of polymerization. Therefore, the nature (hydrophilic or lipophilic) of different compounds present in cocoa can affect the antioxidant capacity results. Furthermore, during processing, native polyphenols can react with proteins, free amino acids, and monosaccharides or polysaccharides to form new compounds with antioxidant properties.^39^

Regarding methylxanthines, FC beans exhibited higher caffeine (2.65 mg/g), and theophylline (0.41 mg/g) levels compared to UC beans (1.16 mg/g and 0.20 mg/g, respectively), while theobromine concentrations were similar in both ecosystems. Previous studies have reported that methylxanthine profiles are associated with the classification of cocoa into Forastero, Trinitario, and Criollo varieties.^40^ In this study, FC showed a theobromine/caffeine ratio of approximately 1.8, which is consistent with values typically observed in Criollo cocoa. In contrast, UC beans presented a theobromine/caffeine ratio of approximately 4.7, a value more commonly associated with Trinitario cocoa.^40^

Caffeine content and the theobromine/caffeine ratio have also been proposed as indicators for classifying cocoa beans based on their geographic origin, with a strong dependence on factors such as plantation altitude.^41^ This approach may also be applicable for distinguishing Amazonian cocoa from different ecosystems. However, further studies are needed to validate these observations and to evaluate the applicability of methylxanthine‐based markers in this context.

### Sensory analyses of the produced chocolate

The sensory evaluation of 70% cocoa chocolates produced from FC and UC beans was conducted. The results detailing the sensory attributes of aroma and flavor are presented in Tables 4 and 5, respectively.

**Table 4:** Sensory attributes of the aroma of chocolates produced from floodplain cocoa (FC) and upland cocoa (UC) beans of the Brazilian Amazon

Aroma	FC	UC	*P*‐Value
Cocoa	7.19 ± 1.28^a^	4.94 ± 1.24^b^	<0.001
Chocolate	7.17 ± 1.03^a^	7.49 ± 1.30^a^	0.540
Acidity	3.41 ± 1.55^a^	1.99 ± 1.7^b^	<0.01
Sweet	4.92 ± 1.78^a^	3.64 ± 1.62^a^	0.180
Fruity	2.42 ± 1.45^a^	2.99 ± 2.06^a^	0.420
Floral	1.64 ± 1.07^b^	2.13 ± 2.09^a^	<0.05
Spices	1.69 ± 0.73^a^	1.47 ± 1.05^a^	0.600
Wood	1.96 ± 0.66^a^	1.64 ± 1.27^a^	0.480
Smoke	0.37 ± 0.52^a^	0.76 ± 0.50^a^	0.120
Animal	0.38 ± 0.44^a^	0.78 ± 0.54^a^	0.090

*Note*: Results expressed as mean ± standard deviation. Different letters in the same line indicate statistical differences (one‐way ANOVA and Tukey's test, *P* < 0.05).

**Table 5:** Sensory attributes of the flavor of chocolates produced from floodplain cocoa (FC) and upland cocoa (UC) beans of the Brazilian Amazon

Flavor	FC	UC	*P*‐Value
Cocoa	7.67 ± 1.33^a^	4.64 ± 1.44^b^	<0.001
Chocolate	7.12 ± 1.26^a^	7.19 ± 1.15^a^	0.850
Acidity	4.46 ± 1.71^a^	3.10 ± 1.49^a^	0.090
Sweet	4.51 ± 1.78^a^	2.88 ± 1.64^b^	<0.01
Fruity	2.62 ± 1.25^a^	2.51 ± 1.18^a^	0.820
Floral	2.03 ± 1.43^a^	1.90 ± 0.98^a^	0.760
Spices	1.53 ± 0.84^a^	1.37 ± 0.65^a^	0.600
Wood	1.38 ± 0.78^a^	1.67 ± 0.84^a^	0.400
Bitterness	4.77 ± 1.52^a^	4.29 ± 1.57^a^	0.520
Astringency	3.36 ± 1.59^a^	3.71 ± 1.35^a^	0.580

*Note*: Results expressed as mean ± standard deviation. Different letters in the same line indicate statistical differences (one‐way ANOVA and Tukey's test, *P* < 0.05).

The chocolate produced from FC beans received intensity scores above seven for cocoa notes in both aroma and flavor. The cocoa attribute is associated with the characteristic flavor of well‐fermented, properly dried, roasted, and defect‐free cocoa beans, which is indicative of successful postharvest processing. The cocoa note is largely influenced by pyrazines, volatile compounds formed through the Maillard reaction during fermentation and drying.^42^

Chocolate notes were similarly intense and relatively high in both chocolates (scores >7). Chocolates produced from FC beans exhibited higher scores for the acidity and sweetness attributes. Acidity is primarily associated with the presence of lactic and acetic acids, which are formed during the fermentation process.^29^ In contrast, chocolates made from UC beans presented more pronounced floral aroma notes. Esters, key contributors to floral notes, are naturally present in fresh cocoa beans but tend to decrease progressively during fermentation.^43^

Both chocolates were evaluated as similar in the attributes of ‘spices,’ ‘woody,’ and ‘fruity,’ which are considered part of the fine‐flavor cocoa profile.^44^ These notes are contributed by a complex mixture of volatile compounds, including alcohols, aldehydes, ketones, esters, terpenoids, and organic acids, which are influenced by factors such as genotype, postharvest practices, and ecosystem conditions.^45^

Bitterness and astringency are mainly associated with the presence of phenolic compounds and methylxanthines in the cocoa beans.^46^ However, the differences observed in the concentrations of these compounds between FC and UC beans were not sufficiently perceptible to result in significant variations in the sensory perception of these attributes. Undesirable attributes (off‐flavors), such as ‘smoky’ and ‘animal,’ were recorded with mean scores below one in both samples, suggesting that these off‐flavor notes have no practical relevance in the sensory quality of the chocolates evaluated.

Sensory analysis of chocolates plays a crucial role in determining product acceptability and, consequently, its market value.^47^ According to the International Cocoa Organization (ICCO), fine cocoa is defined as cocoa that exhibits complex flavor profiles and is free from defects, while also representing significant genetic diversity and historical or cultural heritage.^44^ Although there is no single genetic specification for this category, varieties such as Criollo and Trinitario are predominant among recognized fine cocoas, and other genetic groups, such as Nacional from Ecuador, have also gained increasing international recognition.^43^

Brazil is already recognized as an exporter of fine‐flavor cocoa.^44^ In this context, our results show that chocolates produced from cocoa beans originating from two distinct ecosystems in the Brazilian Amazon exhibited similar sensory profiles, marked by favorable attributes. This suggests that both floodplain and upland environments have the potential to produce cocoa beans capable of meeting the standards of the premium chocolate market.

### Acceptance test

**Figure 4:**
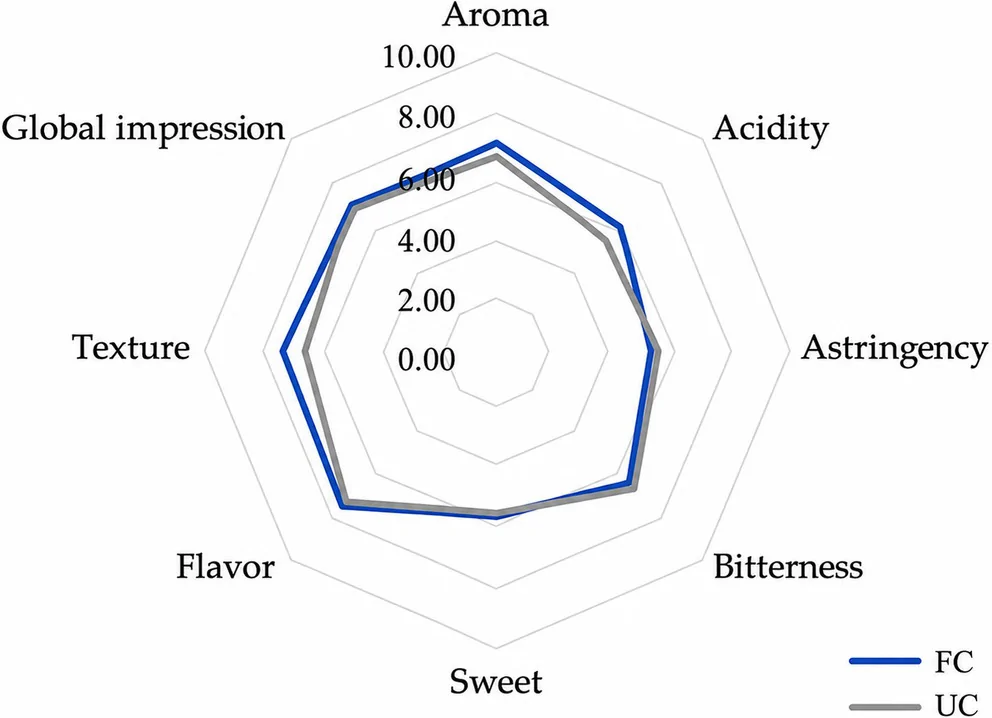
Acceptance test of chocolates produced with floodplain cocoa (FC) beans and upland cocoa (UC) beans from regions of the Brazilian Amazon, conducted with 50 untrained panelists. The evaluation utilized a nine‐point structured hedonic scale with the following descriptors: 9 – ‘liked it extremely,’ 8 – ‘liked it very much,’ 7 – ‘liked it moderately,’ 6 – ‘liked it slightly,’ 5 – ‘neither liked nor disliked,’ 4 – ‘disliked it slightly,’ 3 – ‘disliked it moderately,’ 2 – ‘disliked it very much,’ and 1 – ‘disliked it extremely’. Asterisk (*) indicates significant difference between samples (*P* < 0.05).

The acceptance test was conducted to evaluate the sensory appeal of chocolates produced with beans from each ecosystem. Figure 4 illustrates the tasters' preferences for the FC and UC chocolate samples based on the results of the acceptance test.

Statistical differences (*P* < 0.05) were observed for the attributes of acidity, astringency, and texture between the two samples. The mean acidity scores were 5.96 ± 1.19 for FC and 5.29 ± 1.53 for UC chocolates, with FC showing slightly higher acidity. For the astringency attribute, UC chocolates scored 5.51 ± 0.52, slightly higher than FC (5.11 ± 0.12). However, astringency was evaluated as low to moderate in both samples, a desirable characteristic. The texture attribute showed a clear distinction, with FC chocolates achieving a higher score (7.30 ± 1.01) compared to UC (6.56 ± 0.80).

No significant difference (*P* > 0.05) was observed between the samples for the overall impression attribute, with values of 7.00 ± 1.26 and 7.04 ± 1.43 for FC and UC chocolates, respectively. In a study that evaluated the sensory characteristics of chocolates made with cocoa beans from Bahia, Brazil, the authors reported average overall quality scores ranging from 6.43 to 4.77.^48^

Additionally, overlapping points on the graph for attributes such as aroma, bitterness, sweetness, and flavor suggest similar levels of acceptance for these parameters in both chocolates. The ‘flavor’ attribute was among the most highly rated for both samples, reflecting the quality of the cocoa beans and the adequacy of the processing methods. Subtle differences in flavor perception may be attributed to the balance between sweetness, bitterness, and acidity, which are key elements in the sensory profile of chocolate.^47^ These results are in agreement with those reported in another study that conducted sensory analysis of Amazonian cocoa samples from floodplain areas (Lower Tocantins River region) and upland areas (Trans‐Amazon region).^11^

## CONCLUSIONS

Cocoa fruits from floodplain ecosystems exhibited morphological characteristics consistent with the Forastero group, including an oval shape and thick husks. Although FC beans were smaller and had lower individual mass compared to UC beans, both systems showed adequate fermentation quality. Differences in fiber, proteins, polyphenols, antioxidant capacity, and methylxanthine profiles highlight the compositional variability between ecosystems. Sensory evaluation indicated that chocolates from both origins presented desirable attributes, including floral, fruity, spicy, and woody notes, as well as similar consumer acceptance. These findings demonstrate the potential of Amazonian cocoa from both floodplain and upland systems for product differentiation and the valorization of regionally distinct production systems, reinforcing their economic and social importance to the state of Pará.

## CONFLICT OF INTEREST

The authors declare that they have no known competing financial interests or personal relationships that could have appeared to influence the work reported in this article.

## Data Availability

The data that support the findings of this study are available from the corresponding author upon reasonable request.
